# A Medical Causerie

**Published:** 1916-06-17

**Authors:** 


### A MEDICAL CAUSERIE.

THE proposal to allot all appeals for exemption from military service from medical practitioners to professional committees instead of to the local Tribunals did not pass the House of Commons without discussion and dissent. Some of the members took the opportunity to attack generally the Medical Department of the War Office, and revived the oft-refuted statement that the services of the present officers are ineffective as a result of faulty distribution. Others argued that a special Tribunal was no more needed for doctors than for lawyers, undertakers, grocers, or agricultural labourers. While a third objection, standing in quite a different class, was that no provision was made for an appeal from the decision of the professional committees. In the end the clause was carried by 163 votes to 42. The principal reason for the proposal of course is that the taking of doctors from civil practice has a conspicuous and vital relation to the needs of the community. What are the medical needs of any district can hardly be judged save by a professional Tribunal. Indeed, bearing in mind the needs of the Army on the one hand, and the wants of the community on the other, even a professional committee is likely to find the task both a difficult and a delicate one, and had the matter been left to the local Tribunals, chaos and confusion would have been the inevitable result. Then, if the principle is once granted, the reasons for a right of appeal are difficult to find. If the decision must be in professional hands, such right could only mean a second professional committee, and there is no apparatus in the profession for ranks and orders of authority. The whole position can best be reviewed by a single body whose decisions must be taken less on individual arguments than on the demands of the general situation. As the Military Service Act does not apply to Ireland, the professional committee acting on the other side of St. George's Channel is not formally recognised. But our Irish *confrères* have never been backward in military service, and the present war has proved no exception to a well-established rule.

---

A GOOD example of the critical eye which is directed to the proceedings of the military medical officers is a question recently asked in the House of Commons with a view to discover whether the officers are in the habit of sterilising their hypodermic needles between individual inoculations. Fortunately Mr. Tennant was able to assure the honourable and gallant member who asked the question that there had been no trouble in this respect, and that sterilising the needle after each injection is the routine practice. Public work, of course, implies the chance of public criticism, and the only objection to questions of this order is that they are so often based on vague rumour rather than on precise evidence. Thus they cast undeserved reflections on the Service as a whole. Surely the fairer way would be to draw the attention of the authorities to the particular defaulters rather than to suggest a universal or widespread carelessness.

---

THE attempt to persuade the Otological Section of the Royal Society of Medicine to request the Council of the Society to delete from its roll of membership the names of all corresponding members of German birth for the purpose of expressing disgust at the behaviour of the enemy doctors at Wittenberg camp proved a failure. The original motion was ruled out of order, and a modified proposal to ask the Council to take appropriate steps to voice the general feeling of indignation was rejected. The author of the suggestion has not been content with this decision, and has publicly described the discussion as somewhat "prolonged and pointless"—an opinion which, of course, he is quite entitled to hold. But when he goes on to conclude that because the members who voted against him did not recognise the advisability of a particular medical society taking formal action in the matter they are in favour "of making *no protest whatever* against German barbarity to those typhus-stricken prisoners" he surely exceeds the limits of fair and courteous comment. He frames a large conclusion on very narrow premises, and scarcely shows a capacity to meet defeat with good temper. To say that because members do not approve of his manner of protest they are opposed to every other form is neither good manners nor sound logic.

---

THOUGH the proposal, made in November last, to instruct the Education Committee of the General Medical Council to consider the question of including instruction in professional ethics in the medical curriculum excited a good deal of hostile comment in the Council, the Committee has managed to frame a report which gave general satisfaction. The scheme is far from being a revolutionary one. The Council now announces that such instruction "should be given in the courses of Forensic Medicine and Public Health or otherwise," and adds that "attention should be called to all notices on these subjects issued" by the Council. This grants the principle advocated by Dr. McVail in his original solution, and supported by THE HOSPITAL in a leading article on November 20, 1916. It helps to ensure that no medical student shall leave his school without having had some opportunity of realising some of the special ethical responsibilities which weigh upon the practitioner in relation to his patients, to his fellows, and to public bodies. Recent cases which have come before the Council show conclusively that some members of the profession get themselves into false positions, not so much by deliberate ill-doing as by a failure to recognise that easy good nature towards individuals is less important than attention to rules which have the general welfare as their justification. A little vigorous teaching in the early stage may be expected to avoid much trouble at a later date.

